# Supplementary material for: Neuropeptide Neuromedin B does not alter body weight and glucose homeostasis nor does it act as an insulin-releasing peptide
Source: Sci Rep. 2022 Jun 7;12:9383. doi: 10.1038/s41598-022-13060-0 (PMC9174263; doi:10.1038/s41598-022-13060-0)
Supplement: Supplementary file 1 — Supplementary Information 1. [file 41598_2022_13060_MOESM1_ESM.pdf]

Supplementary Figure 1

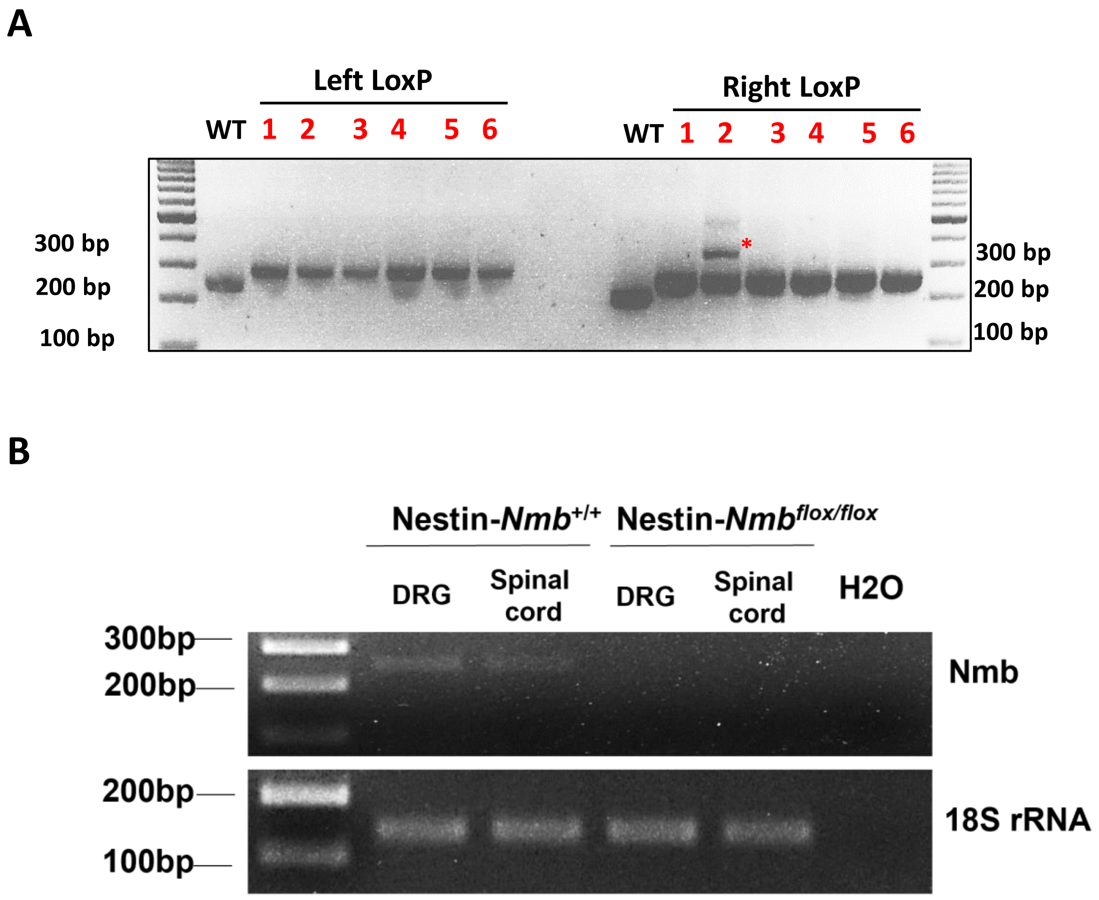

Supplementary Figure 1:

A) Full image version of the validation for the correct insertion of LoxP sites by PCR with primers placed in the genomic region outside of the repair ssDNA template. Each band represents a PCR product from an individual animal, N=7. Asterisk denotes an unspecific band in one of the PCR reactions. **B)** Validation of Nmb deletion by the RT-PCR in the dorsal root ganglia (DRG) and the spinal cord.
