# Supplementary material for: Neuropeptide Neuromedin B does not alter body weight and glucose homeostasis nor does it act as an insulin-releasing peptide
Source: Sci Rep. 2022 Jun 7;12:9383. doi: 10.1038/s41598-022-13060-0 (PMC9174263; doi:10.1038/s41598-022-13060-0)
Supplement: Supplementary file 2 — Supplementary Information 2. [file 41598_2022_13060_MOESM2_ESM.pdf]

Supplementary Figure 2

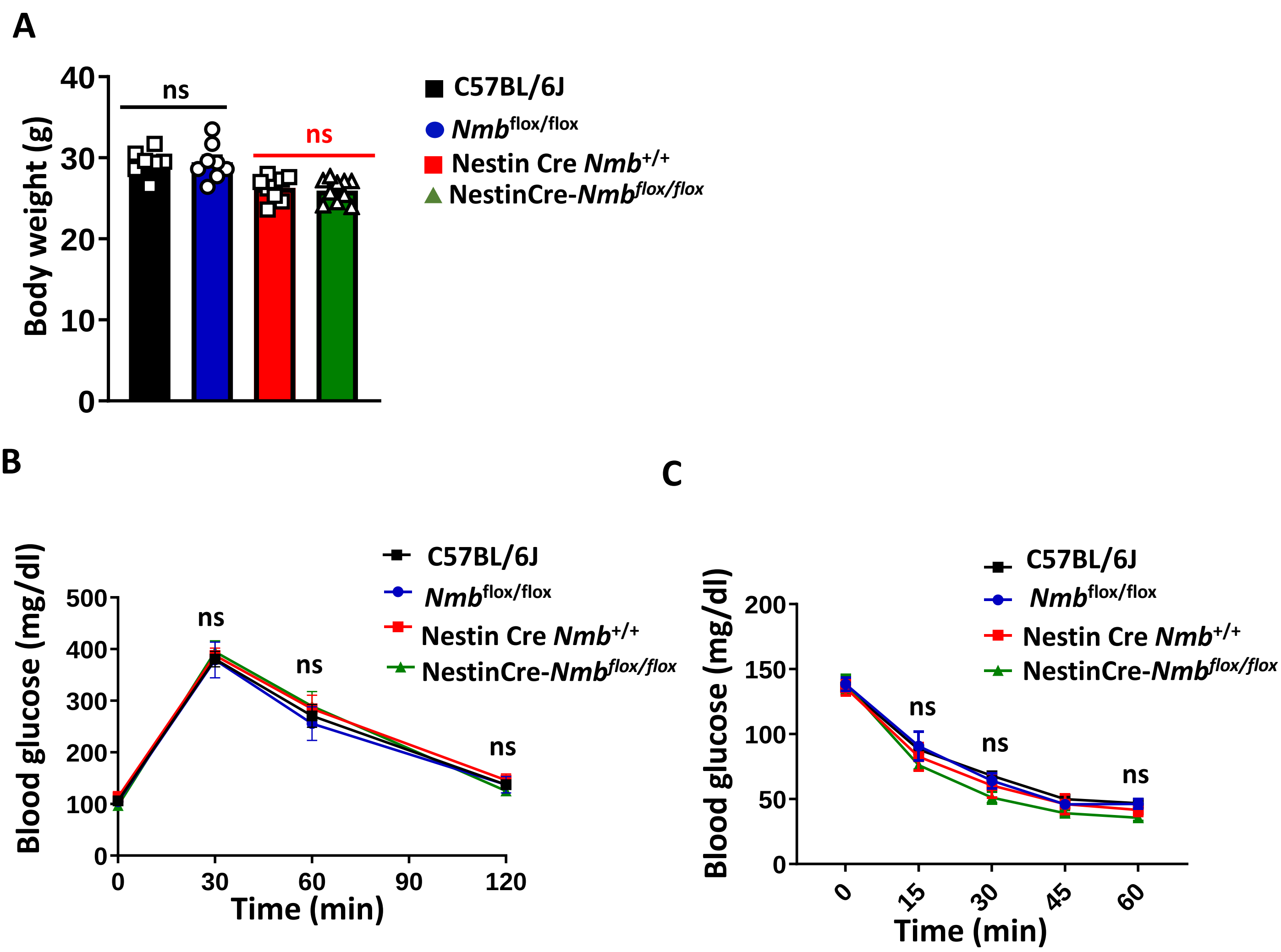

Supplementary Figure 2:

A) Body weight of 6 month old Nmb$^{flox/flox}$ and C57BL/6J mice on standard diet. Each dot represents individual mice. **(B-C)** Intraperitoneal glucose tolerance test (IP GTT) and intraperitoneal insulin tolerance test (ITT) of 6 month old Nmb$^{flox/flox}$ and C57BL/6J mice on standard diet. n=6-8 mice per group. Data are shown as means ± SEM. For statistical analysis, unpaired Student t-test and two-way ANOVA followed by Bonferroni’s post-hoc test. ns- non significant;
