# Supplementary figures and images for: Neuropeptide Neuromedin B does not alter body weight and glucose homeostasis nor does it act as an insulin-releasing peptide
Source: Sci Rep. 2022 Jun 7;12:9383. doi: 10.1038/s41598-022-13060-0 (PMC9174263; doi:10.1038/s41598-022-13060-0)

# Supplementary Figure 3

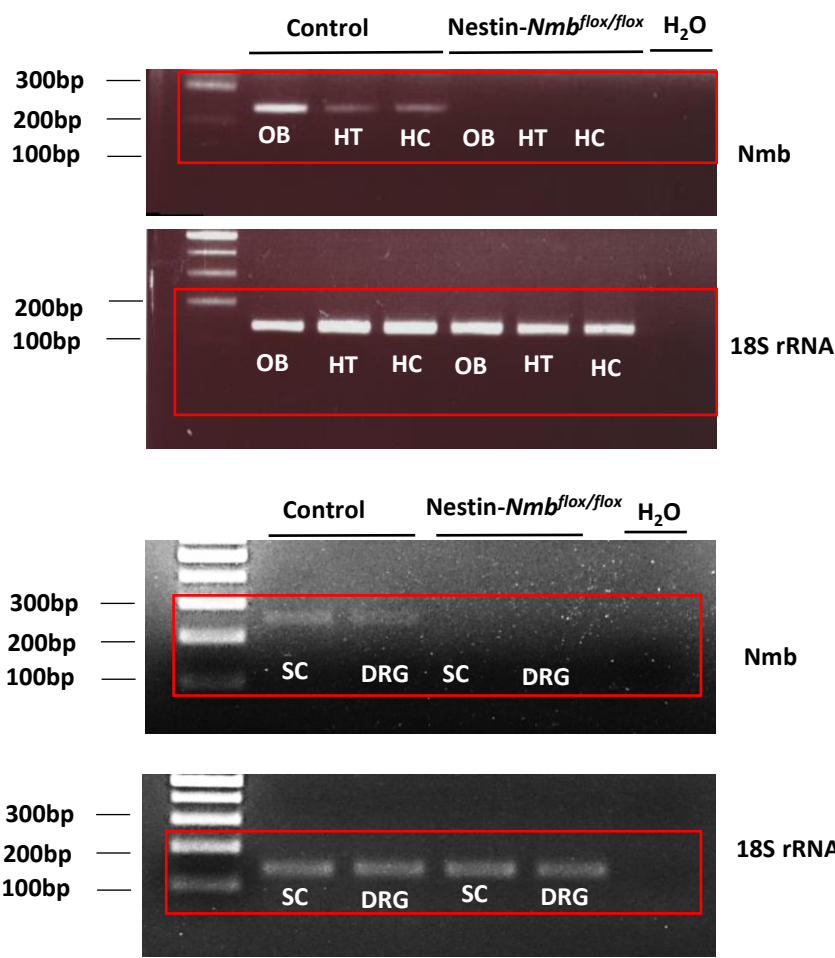

## Supplementary Figure 3:

Uncropped gel images used in the manuscript

Supplement: Supplementary file 3 — Supplementary Information 3. [file 41598_2022_13060_MOESM3_ESM.pdf]
